# Timeline of heparin‐induced thrombocytopenia seroconversion in serial plasma samples tested using an automated latex immunoturbidimetric assay

**DOI:** 10.1111/ijlh.13031

**Published:** 2019-05-03

**Authors:** Theodore E. Warkentin, Jo‐Ann I. Sheppard, James W. Smith, Donald M. Arnold, Ishac Nazy

**Affiliations:** ^1^ Department of Pathology and Molecular Medicine, Michael G. DeGroote School of Medicine McMaster University Hamilton Ontario Canada; ^2^ Department of Medicine, Michael G. DeGroote School of Medicine McMaster University Hamilton Ontario Canada; ^3^ McMaster Centre for Transfusion Research Hamilton Ontario Canada; ^4^ Hamilton Regional Laboratory Medicine Program Hamilton Health Sciences Hamilton Ontario Canada

**Keywords:** heparin‐induced thrombocytopenia, HIT antibody seroconversion, latex immunoturbidimetric assay, serotonin‐release assay (SRA), SRA‐negative HIT

## Abstract

**Introduction:**

HIT is caused by platelet‐activating IgG that recognize multimolecular PF4/heparin complexes. HIT antibodies are generally detectable by PF4‐dependent enzyme immunoassay (EIA) and by platelet serotonin‐release assay (SRA) at the beginning of the HIT‐related platelet count fall. We determined whether an automated immunoassay for HIT, the latex immunoturbidimetric assay (LIA), also detects antibodies early during the course of HIT. The LIA was also used to evaluate a patient with putative SRA‐negative HIT.

**Methods:**

We evaluated the timing and magnitude of LIA reactivity in serial plasma samples obtained from 19 SRA‐positive patients (17 with abnormal platelet count changes indicating HIT; two with subclinical seroconversion) and one putative SRA‐negative HIT patient, all obtained from patients who participated in a clinical trial of heparin thromboprophylaxis. We determined LIA status at the onset of the HIT‐related platelet count fall.

**Results:**

The LIA was positive in all 19 SRA‐positive patients (median value, 7.3 U/mL [range, 1.2‐35.5]; cutoff, 1.0 U/mL); for all 13 evaluable patients for whom an informative plasma sample was available at (or shortly before) the onset of the HIT‐related platelet count fall, LIA reactivity was positive. Heterogeneity in seroconversion using the LIA was observed; some patients exhibited gradual increases in reactivity, whereas other patients showed rapid increase in reactivity over a few days. The single clinical trial patient who met clinical‐pathological criteria for “SRA‐negative HIT” tested LIA‐positive.

**Conclusion:**

The LIA detects HIT antibodies at the beginning of the HIT‐associated platelet count fall. The LIA was also positive in a patient with SRA‐negative HIT.

## 1 INTRODUCTION

Immune heparin‐induced thrombocytopenia (HIT) is caused by platelet‐activating antibodies that recognize multimolecular complexes of a cationic protein, platelet factor 4 (PF4), bound either to heparin or to platelet‐associated polyanions.^1^, ^2^, ^3^ An important feature of HIT is the high sensitivity (≥97%) of PF4‐dependent immunoassays for detecting pathogenic antibodies,^4^, ^5^ in contrast to the much lower sensitivity of platelet antibody testing for other immune‐mediated thrombocytopenic disorders, such as idiopathic thrombocytopenic purpura^6^, ^7^, ^8^ or “classic” (non‐HIT) drug‐induced immune thrombocytopenia.^9^, ^10^ We have previously reported that PF4‐dependent enzyme immunoassays (EIAs) and the platelet serotonin‐release assay (SRA) are generally positive at the onset of the HIT‐related platelet count fall, including during the early phase of the platelet count decline when HIT would not even be suspected.^11^, ^12^ We have proposed^13^ that the high sensitivity of serum/plasma‐based assays for HIT antibodies may have implications for the pathogenesis of HIT; for example, significant levels of free (unbound) HIT antibodies might be required to produce the dynamic conditions essential to form multimolecular PF4‐polyanion complexes on platelet surfaces needed to engage and cross‐link platelet FcγIIa receptors, resulting in Fc receptor‐mediated platelet activation characteristic of HIT.^14^, ^15^, ^16^

We capitalized on the availability of archived plasma samples from a clinical trial of heparin thromboprophylaxis to examine whether early detectability of HIT antibodies is also seen with an automated, rapid immunoassay, known as the latex immunoturbidimetric assay or LIA. To our knowledge, studies using serial plasma samples during acute seroconversion have not been reported using this assay. The LIA is performed on a coagulation instrument (ACL TOP^®^ Family, Instrumentation Laboratory), and thus, plasma, rather than serum, is used for testing.^17^ As most testing for HIT is performed using serum, the availability of these well‐characterized plasma samples provided us a unique opportunity to evaluate LIA reactivity associated with HIT seroconversion. Given the potential for increased use of this assay to provide real‐time, on‐demand testing for HIT antibodies^18^, ^19^—including incorporation into real‐time Bayesian diagnostic analysis^20^—we sought to determine the changes in LIA reactivity during acute seroconversion and, particularly, whether assay positivity occurs during the earliest phase of HIT, at a time when the platelet count has begun to decline but usually too early for a diagnosis of HIT to be contemplated.

During the study, we became aware of one study patient who had a high clinical probability of HIT, and who tested EIA‐positive. However, this patient had tested negative in the platelet serotonin‐release assay (SRA) and, thus, was previously considered by our group not to have had HIT. In recent years, the concept of SRA‐negative HIT has been proposed,^21^, ^22^, ^23^ in which subthreshold levels of heparin‐dependent platelet‐activating antibodies (by SRA) can be detected using modifications to platelet activation assays such as addition of PF4^24^, ^25^ aimed to increasing test sensitivity. Therefore, we investigated whether this patient met clinical and laboratory criteria for SRA‐negative HIT. As serial plasma samples were available for this patient, we also studied the LIA seroconversion profile of this patient with putative SRA‐negative HIT.

## 2 PATIENTS AND METHODS

### 2.1 Archived blood samples from clinical trial

Archived plasma samples were available from a clinical trial of heparin thromboprophylaxis.^26^ We have previously tested these plasma samples by SRA,^27^ in‐house IgG‐specific EIA,^28^ commercial IgG‐specific EIA (LIFECODES PF4 IgG assay; Immucor GTI Diagnostics),^29^ and commercial polyspecific EIA that detects antibodies of IgG, IgA, and IgM classes (LIFECODES PF4 Enhanced assay; Immucor GTI Diagnostics),^30^ to elucidate certain clinical and laboratory features of HIT^11^, ^12^, ^31^, ^32^; however, we have not previously utilized these samples for LIA testing, nor for investigating patients with putative SRA‐negative HIT. In this study, we focused on examining LIA seroconversion among SRA‐positive patients, as well as determining whether any clinical trial patients met criteria for SRA‐negative HIT. As described subsequently, we therefore identified all available archived patient plasma samples from the aforementioned thromboprophylaxis clinical trial in which patients were either known to have previously tested SRA‐positive or had tested SRA‐negative/EIA‐positive but who a clinical picture suggesting a plausible diagnosis of HIT (discussed later in section, Investigations for SRA‐negative HIT).

Permission was received from the Hamilton Integrated Research Ethics Board to perform these studies (#1288‐T).

### 2.2 Definitions of HIT

Patients from the clinical trial met one of the following previously published definitions for HIT 12, 31, 32: (a) classic (standard) definition of thrombocytopenia, that is, a platelet count falls to less than 150 × 10^9^/L that began five or more days after starting heparin^31^; (b) proportional definition of thrombocytopenia, that is, platelet count fall of at least 50%^32^ or 30.0% to 49.9%^12^ from the postoperative peak that began five or more days after starting heparin; or (c) blunted platelet count recovery, that is, a platelet count that did not show the expected platelet count rise during the second postoperative week, and which also fell below the bounds of an appropriate control antibody‐negative patient population with similar baseline (preoperative) platelet counts.^12^ A fourth group of SRA‐positive patients identified from this clinical trial comprised those patients who exhibited none of the aforementioned pathological platelet count profiles, but in whom SRA seroconversion was documented; these patients were classified as having “subclinical seroconversion”.

### 2.3 Patient samples studied

Although many of the archived plasma samples had been expended during these and other studies, we were able to evaluate remaining samples available from each of the following patients groups for this study: (a) classic (standard) definition of thrombocytopenia, n = 5; (b) proportional thrombocytopenia, n = 8 (with six patients meeting >50% fall and two patients with 30.0%‐49.9% platelet count fall, and with none of these eight patients attaining a platelet count nadir less than 150 × 10^9^/L); (c) blunted platelet count recovery, n = 4; and (d) subclinical seroconversion, n = 2. In addition, we had available serial plasma samples from a patient with (e) putative SRA‐negative HIT (see next section).

### 2.4 Investigations for SRA‐negative HIT

We used the following criteria for SRA‐negative HIT^24^: (a) high probability 4Ts score and PF4‐dependent EIA > 1.00 optical density (OD) units or intermediate probability 4Ts score and PF4‐dependent EIA > 2.00 units; and (b) SRA‐negative (including on repeat testing) but positive in a PF4‐enhanced platelet activation assay. We used a PF4‐enhanced SRA^25^ in which the standard SRA^27^ is performed with the addition of PF4 (50, 100 µg/mL) rather than unfractionated heparin. We defined a positive PF4‐SRA as one that showed increase by 20% or more of serotonin release in the presence of PF4 (vs the buffer control), that is, the same magnitude of increase in serotonin release that defines a positive test in the (classic) SRA.^27^ To evaluate thromboprophylaxis trial patients for possible SRA‐negative HIT, we identified all EIA‐positive, SRA‐negative patients who exhibited 30% or greater declines in the platelet count beginning on or after day 5 of heparin therapy.

### 2.5 Latex Immunoturbidimetric Assay (LIA)

Although classified as an immunoassay, the LIA differs from most other immunoassays, such as EIAs, in that the presence of anti‐PF4/polyanion antibodies within patient plasma result in the inhibition of agglutination of KKO‐coated nanoparticles to which PF4/polyvinylsulfonate (PVS) complexes have been added, through competition with KKO, a HIT antibody‐mimicking monoclonal antibody (PVS is a polyanion that mimics heparin in being able to produce conformational changes in PF4 that result in the formation of the HIT antigen[s]^30^). Thus, the LIA has been called a "functionalized immunoassay,"^19^ both to reflect its unique methodology, as well as the observation that its diagnostic specificity is intermediate between the highly specific functional assays (eg, SRA) and the less specific PF4‐dependent EIAs.^20^

The LIA [HemosIL^®^ HIT‐Ab_(PF4‐H)_] was performed using the ACL TOP^®^ 500 CTS instrument (Instrumentation Laboratory) following the manufacturer's recommendations. As per the manufacturer, a test result of 1.0 U/mL or greater is considered positive. The test range of the LIA is from 0 to 5.7 U/mL. When positive results occur above this range, the test is automatically rerun, after making an onboard 1/4 dilution, which expands the measurement range to 16.0 U/mL. However, we modified our test definition to perform additional automated onboard dilutions (twofold, up to 1/32), which allowed for a (calculated) positive result as high as 182.4 U/mL. As previously noted, all studies were performed using plasma anticoagulated with sodium citrate.

### 2.6 Timing of LIA vs EIA‐GAM seroconversion

For patients with available/informative blood samples, we compared the timing of seroconversion between the LIA and the commercial polyspecific EIA (EIA‐GAM), determining whether seroconversion occurred earlier for one or the other assay, or occurred on the same postoperative day.

### 2.7 Comparison with LIA reactivities from other studies

We compared the LIA reactivities of 20 patients from our study with those of 156 consecutive HIT patients identified in our institution in a recent study of the LIA for diagnosis of HIT,^20^ as well as an additional 23 consecutive patients with HIT more recently recognized at our institution (Hamilton General Hospital). This comparison was performed to determine if the distribution of positive LIA results differed substantially between the patients studied in our thromboprophylaxis trial (all of whom underwent hip replacement) vs predominantly nonorthopedic surgery patients (the Hamilton General Hospital is a regional trauma center that also has large medical, cardiology, cardiac surgery, and vascular surgery patient populations).

## 3 RESULTS

### 3.1 LIA reactivities in SRA‐positive archived plasma samples

Table 1 summarizes the results of LIA testing for 19 patients with archived plasma samples from the orthopedic surgery thromboprophylaxis trial who tested SRA‐positive, as well as a 20th patient who was investigated for possible SRA‐negative HIT. Each patient is classified as per the applicable definition of HIT. For each patient, the maximal LIA result is shown; along with the postoperative day (POD), the plasma sample was obtained. The LIA was positive in all 19 SRA‐positive patients (median value, 7.3 U/mL [range, 1.2—35.5]; cutoff, 1.0 U/mL). In addition, the LIA was also positive in the patient recognized as having SRA‐negative HIT (discussed subsequently in section “SRA‐negative HIT”).

**Table 1:** LIA reactivity in 19 SRA‐positive patients and 1 additional patient with SRA‐negative HIT

Patient Age/Sex	HIT definition	Platelet count nadir, ×10^9^/L (% fall)	Maximal LIA result, U/mL, postoperative day (POD)	Result at beginning of platelet count fall					Figure showing corresponding serial serological data
				POD	LIA (U/ml)	SRA (% release)	EIA‐GAM (U/mL)	EIA‐G (U/mL)	
63F	Classic (<150)	28 (84.9%)	35.5 (POD8)	5	1.9	69	2.08	0.74	Figure 1A; Figure 2A
64F	Classic (<150)	22 (93.1%)	9.5 (POD9)	7	7.8	100	1.65	1.27	Figure 1B
65F	Classic (<150)	34 (90.1%)	7.3 (POD12)	7	1.9	81	2.74	1.64	Figure 1C
73F	Classic (<150)	75 (83.0%)	2.0 (POD10)	7	NA	90	2.21	1.09	Figure 1D
72M	Classic (<150)	71 (82.6%)	1.8 (POD13)	6	NA	NA	NA	NA	No
60F	Proportional > 50%	329 (53.7%)	17.7 (POD9)	9	17.7	89	2.22	1.41	Figure 1E
57M	Proportional > 50%	297 (57.9%)	18.6 (POD12)	10	18.1	100	2.80	1.90	Figure 1F; Figure 2F
62F	Proportional > 50%	169 (70.8%)	12.3 (POD13)	8	4.0^a^	53	1.73	0.82	Figure 1G
87F	Proportional > 50%	161 (58.0%)	5.7 (POD13)	7	1.1	97	2.56	1.42	Figure 1H; Figure 2H
74F	Proportional > 50%	197 (61.1%)	1.5 (POD9)	11	1.1^b^	54	2.05	1.20	Figure 1I
67F	Proportional > 50%	159 (58.4%)	10.9 (POD8)	6	NA	NA	NA	NA	No
64F	Proportional > 30%	204 (34.8%)	10.4 (POD12)	9	9.0	NA	2.59	1.49	Figure 1J
74M	Proportional > 30%	282 (31.9%)	1.9 (POD7)	8	1.7	83	1.40	1.03	Figure 1K
45F	Blunted recovery	448 (17.8%)	17.1 (POD9)	10	17.1^b^	82	2.73	1.69	Figure 1L; Figure 2L
74M	Blunted recovery	212 (5.4%)	2.7 (POD13)	9	1.0^b^	54	1.14	0.95	Figure 1M
79F	Blunted recovery	433 (19.5%)	1.2 (POD14)	11	NA	69	2.73^b^	1.81^b^	No
65M	Blunted recovery	237 (22.3%)	2.4 (POD12)	11	NA	72	1.45	0.91	No
57M	Subclinical SRA seroconversion^c^	No platelet fall	32.4 (POD8)^d^	NA	32.4^d^	84^d^	2.32^d^	1.78^d^	No
77M	Subclinical SRA seroconversion^e^	No platelet fall	1.3 (POD10)	NA	NA	83^d^	1.67^d^	0.98^d^	No
72F	Proportional (>50%); SRA‐negative HIT	159 (58.9%)	5.5 (POD8)	8	5.5	37 (PF4‐SRA)	0.83	1.25	Figure 3

Nine of the 20 patients shown in the Table developed HIT‐associated thrombosis (classic, n = 3; proportional, n = 3; blunted recovery, n = 1; subclinical seroconversion, n = 1; and the patient with SRA‐negative HIT).

EIA‐G, enzyme immunoassay that detects IgG class antibodies (McMaster in‐house EIA); EIA‐GAM, enzyme immunoassay that detects IgG, IgA, and/or IgM class antibodies (polyspecific); F, female; HIT, heparin‐induced thrombocytopenia; LIA, latex immunoturbidimetric assay; M, male; NA, blood sample not available; PF4‐SRA, serotonin‐release assay performed with different concentrations of PF4 rather than heparin; POD, postoperative day; SRA, serotonin‐release assay; U, units.

a Sample obtained 2 d prior to the onset of platelet count fall.

b Sample obtained 1 d prior to the onset of platelet count fall.

c No evidence of HIT by platelet count fall or thrombosis, but the patient did present approximately 2 mo later with symptomatic pulmonary embolism.

d First day that positive SRA was observed.

e Venogram report showed “valve clot in superficial femoral vein”.

For 13 of the 20 patients shown in Table 1, a LIA result was available from a plasma sample obtained either on the same day the platelet count began to fall (n = 9), or if not available on the first day of platelet count fall, either one (n = 3) or two (n = 1) days prior to the onset of platelet count fall; for the other seven patients, the available plasma samples were from days after the onset of the HIT‐related platelet count fall had already occurred, and thus, LIA status at the onset of the HIT‐related platelet count fall could not be determined. We found that for all 13 evaluable patients, the LIA test was positive at (or shortly before) the onset of the HIT‐related platelet count fall (median value, 4.0 U/mL [range, 1.0—18.1]), as were the other assays we performed (SRA, two different EIAs). Thus, as we have reported previously for both the EIA^11^ and the SRA,^12^ the LIA also is positive at the beginning of the HIT‐related platelet count fall.

### 3.2 LIA Seroconversion

**Figure 1:**
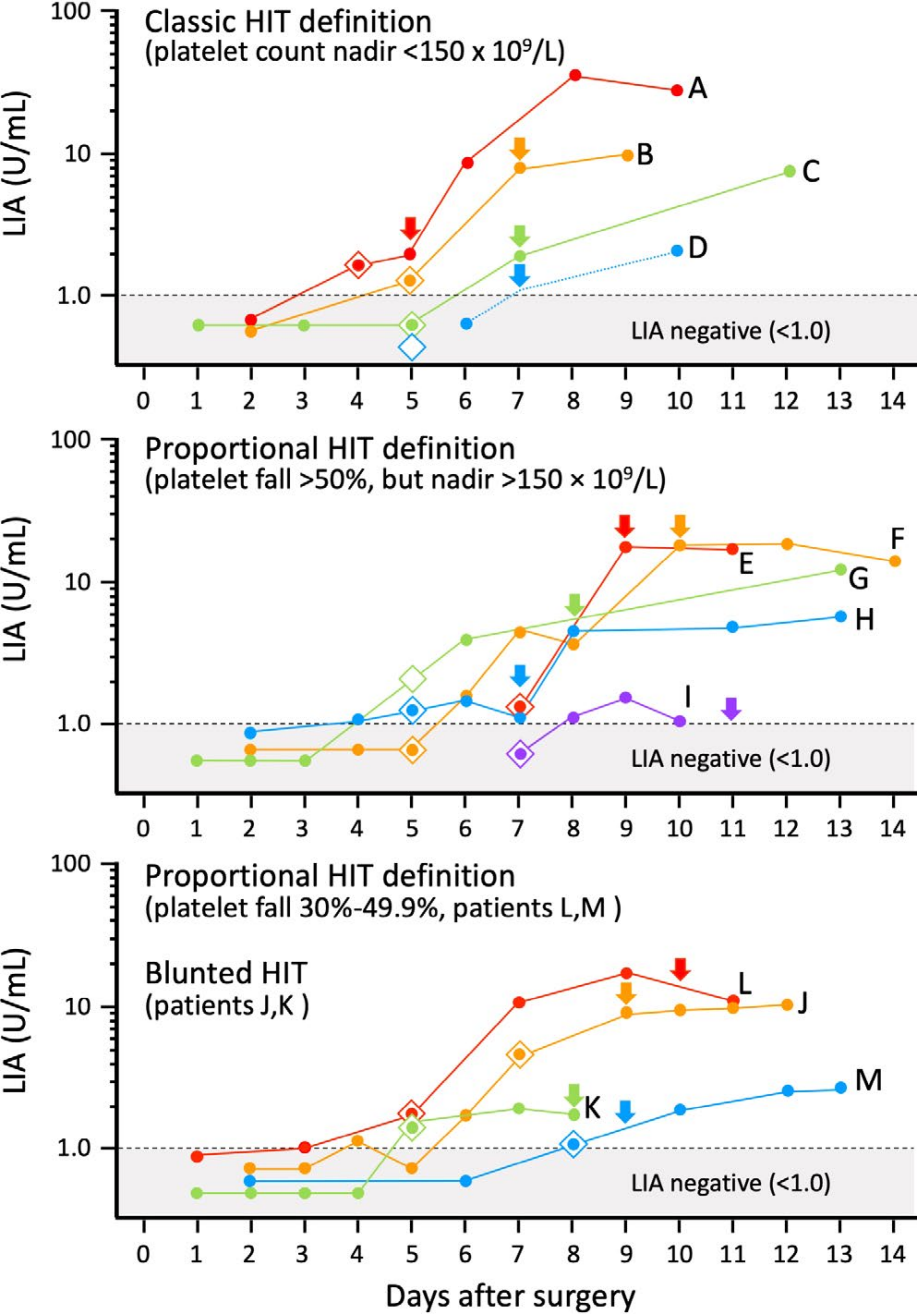
LIA seroconversion in 13 patients with HIT. For each patient, the corresponding colored arrow indicates the day of onset of the HIT‐related platelet count fall, and the corresponding colored diamond symbol indicates the day of seroconversion by EIA‐GAM. The top part of the graph shows four patients (A through D) who met the classic definition of HIT; the middle part of the graph shows five patients (E through I) who met the proportional (>50%) definition of HIT (but without developing a platelet count nadir <150 × 10^9^/L); the bottom part of the graph shows 4 patients (J through M) who had less marked declines in platelet count, either proportional (30%‐49.9%) or with blunted platelet count recovery. For all 13 patients shown in the graph, the LIA was positive at the beginning of the HIT‐related platelet count fall. Please see text for information regarding the relative timing of seroconversion by EIA‐GAM vs seroconversion by LIA. All patient designations are per Table 1. EIA‐GAM, enzyme immunoassay that detects IgG, IgA, and/or IgM class antibodies (polyspecific); HIT, heparin‐induced thrombocytopenia; LIA, latex immunoturbimetric assay; U, units

Figure 1 shows serial sample LIA reactivities of 13 SRA‐positive patients in whom we were able to ascertain LIA seroconversion in relation to the beginning of the HIT‐related platelet count declines (the arrows indicate the onset of HIT). The upper panel shows LIA reactivities for the four patients who met the classic definition of HIT, that is, platelet count falls to less than 150 × 10^9^/L. The middle panel shows LIA reactivities for the five patients who exhibited a large proportional drop in platelet count (>50%) but whose platelet count did not fall below 150 × 10^9^/L. Finally, the lower panel shows serial LIA reactivities in the four seroconverting patients with less marked decreases in platelet count, either proportional (30%‐49.9% platelet count fall) or blunted platelet count recovery patterns. Interestingly, for all patient groups, there was wide variability in the degree of LIA reactivity, with some patients in all three groups attaining high LIA reactivities (>10 U/mL), yet other patients having results not much higher than the assay cutoff.

For these 13 patients, there was a median of six available samples per patient (range, 2‐10 samples). The arrow indicates the day of onset of the HIT‐related platelet count fall. For all 13 patients, positive LIA status was found at the time (or shortly before) the platelet count fall had begun.

### 3.3 Timing of LIA vs EIA‐GAM seroconversion

Figure 1 also shows the day of seroconversion by EIA‐GAM (indicated by the diamond symbol color‐matched for each patient). For four patients (C, D, F, and I), EIA‐GAM seroconversion occurred before LIA seroconversion, as shown by the corresponding diamond symbols located below the LIA 1.0 cutoff line. For four other patients (H, J, L, as well as the SRA‐negative HIT patient discussed subsequently [not shown on Figure 1]), LIA seroconversion occurred before EIA‐GAM seroconversion. For the remaining patients, seroconversion either occurred on the same day (patients A, B, E, K, M) or was indeterminate (patient G).

### 3.4 Seroconversion‐clinical timelines: representative cases

**Figure 2:**
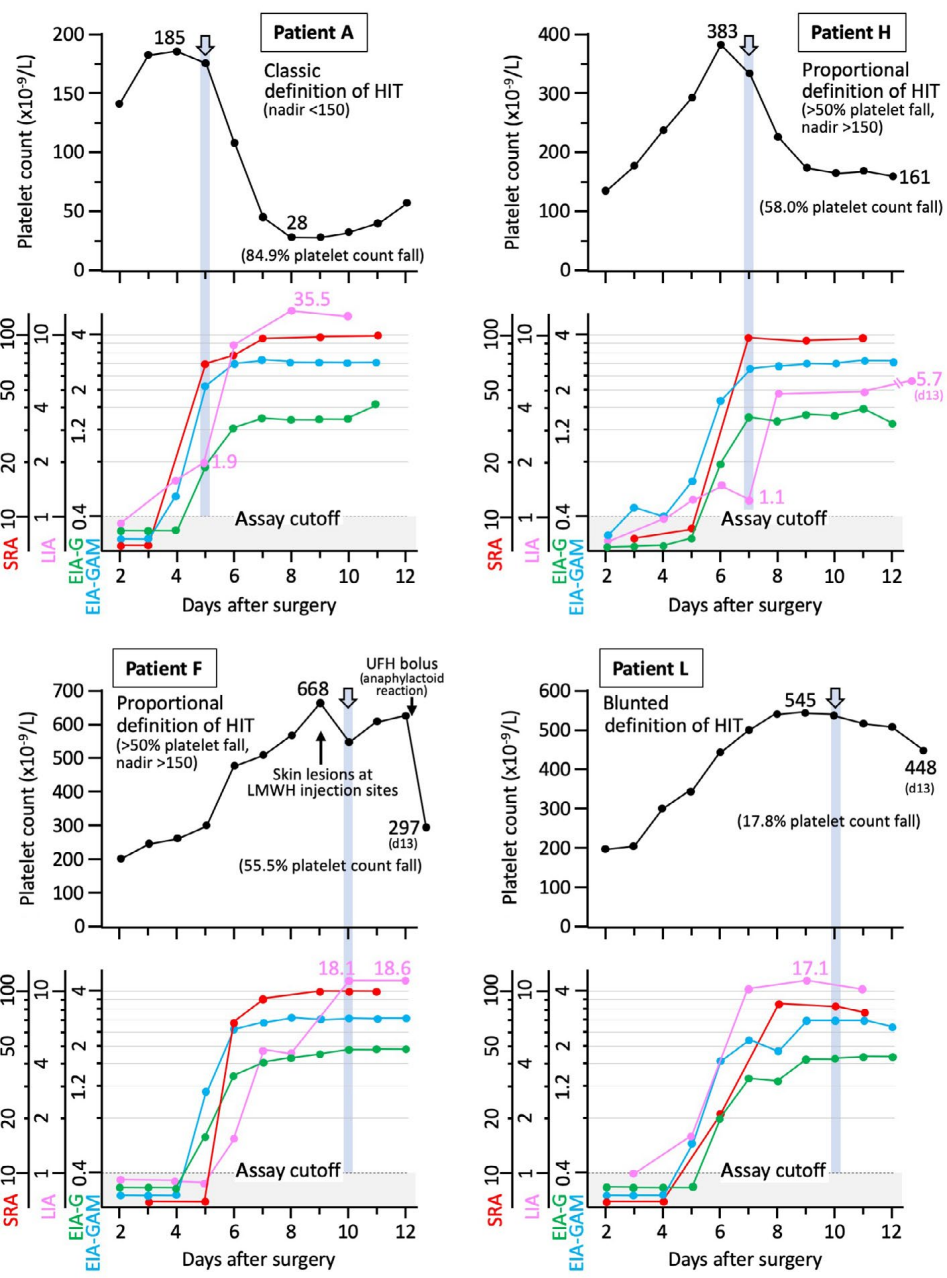
Four patients (A, F, H, L) showing the inter‐relationship between HIT antibody seroconversion (by four assays—SRA, EIA‐IgG, EIA‐GAM, and LIA) and associated platelet count changes. EIA‐G, enzyme immunoassay that detects IgG antibodies (McMaster in‐house EIA); EIA‐GAM, enzyme immunoassay that detects IgG, IgA, and/or IgM class antibodies (polyspecific); HIT, heparin‐induced thrombocytopenia; LIA, latex immunoturbidimetric assay; LMWH, low molecular weight heparin; SRA, serotonin‐release assay; UFH, unfractionated heparin

Figure 2 shows four representative patients for whom we show the LIA and EIA seroconversion in relation to platelet count values. Of the four patients shown, one met the classic definition of thrombocytopenia (patient A in Figure 1), two patients met proportional decreases in platelet count of at least 50% but without a fall to less than 150 × 10^9^/L (patients F and H in Figure 1), and one patient exhibited a blunted platelet count recovery (patient L in Figure 1, lower panel). The figure shows that for all four tests (SRA, EIA‐GAM, EIA‐G, LIA), results were positive at the time the platelet count began to fall (as indicated by the arrows and associated vertical light blue shading).

### 3.5 SRA‐negative HIT

**Figure 3:**
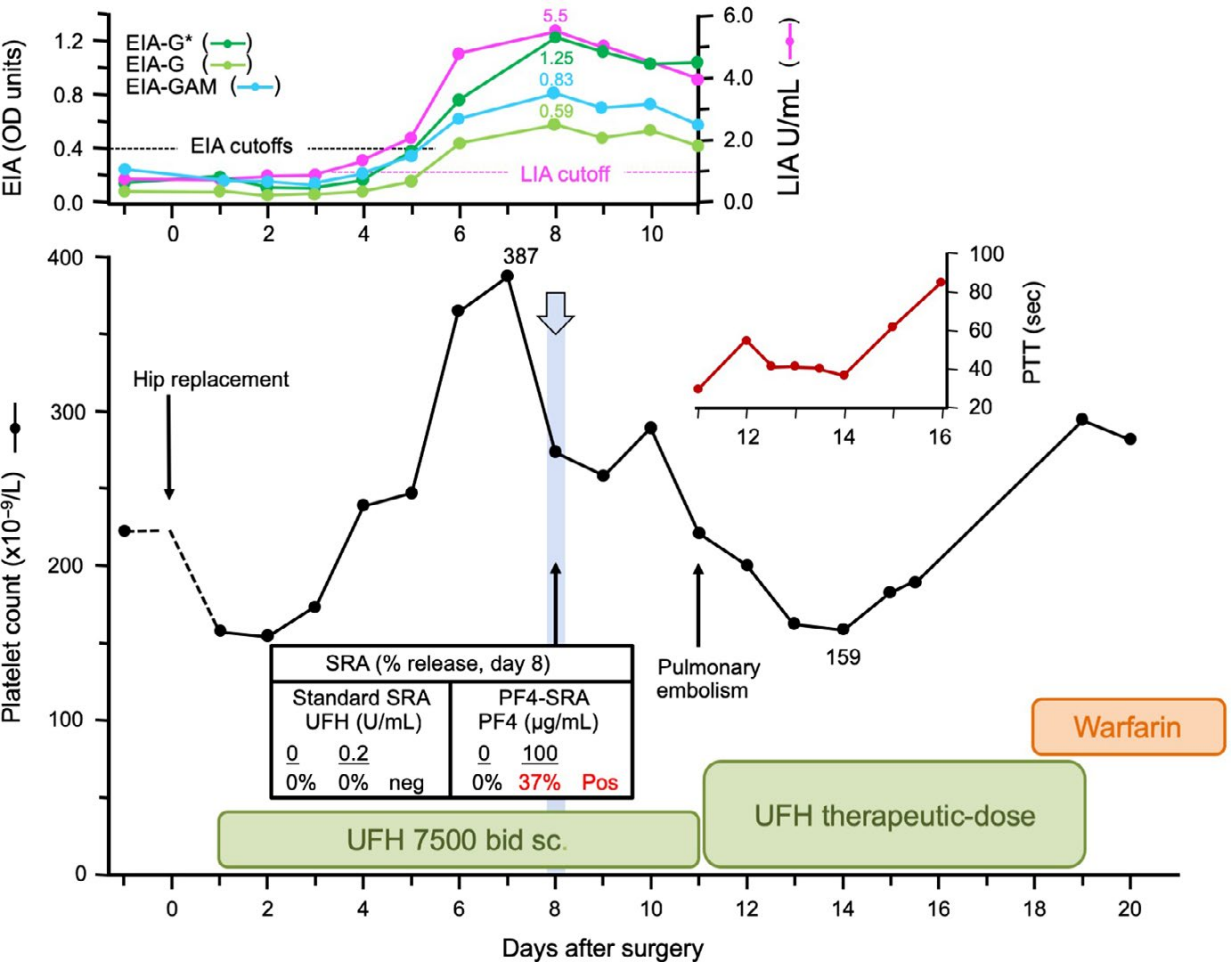
Patient with SRA‐negative HIT. Serial platelet counts show a platelet count fall from 387 × 10^9^/L to 159 × 10^9^/L (58.9% fall); pulmonary embolism was diagnosed on postoperative day 11, and the patient was treated with therapeutic‐dose UFH. HIT was not clinically suspected. During subsequent HIT‐related research studies, the patient was not believed to have had HIT, as the SRA was negative. However, during the evaluation of the LIA, the high probability clinical picture, as well as the seroconversion by four immunoassays (two IgG‐specific EIAs, with the McMaster in‐house EIA indicated by the asterisk [*], a polyspecific EIA, as well as the LIA), prompted investigations for SRA‐negative HIT. We found that the day 8 plasma sample yielded a positive PF4‐SRA (37% serotonin release at 100 µg/mL PF4; 0% release with the addition of 100 U/mL heparin), confirming a diagnosis of SRA‐negative HIT. EIA‐G, enzyme immunoassay that detects IgG class antibodies; EIA‐GAM, enyzme immunoassay that detects IgG, IgA, and/or IgM class antibodies (polyspecific); LIA, latex immunoturbidimetric assay; PF4, platelet factor 4; PTT, partial thromboplastin time; sec, seconds; SRA, serotonin‐release assay; UFH, unfractionated heparin; U, units

Figure 3 summarizes the case of a patient who met the criteria for SRA‐negative HIT. This patient had a high probability for HIT based on a high 4Ts score: 2 points for thrombocytopenia (platelet count fall of 58.9% from 387 to 159 × 10^9^/L [nadir]), 2 points for day 7 timing of onset of thrombocytopenia, 2 points for thrombosis (pulmonary embolism), and 2 points for other explanation for thrombocytopenia not identified. As this patient had a positive EIA that exceeded 1.00 OD units (1.25 OD units per McMaster IgG‐specific EIA), the patient met the combined clinical and laboratory criteria for putative SRA‐negative HIT, that is, high 4Ts score, PF4‐dependent EIA > 1.00 OD units, and SRA‐negative status.^24^ Despite the patient's plasma samples testing repeatedly SRA‐negative, there was a positive test in the PF4‐SRA (30.3% and 37.2% at 50 and 100 µg/mL PF4, respectively, with reactivity at buffer control of 0%), indicating the presence of subthreshold levels of platelet‐activating antibody by standard SRA. This patient also exhibited clear seroconversion by LIA (from 0.8 to 5.5 U/mL [maximal reactivity]).

### 3.6 Distribution of trial results vs comparator HIT populations

**Figure 4:**
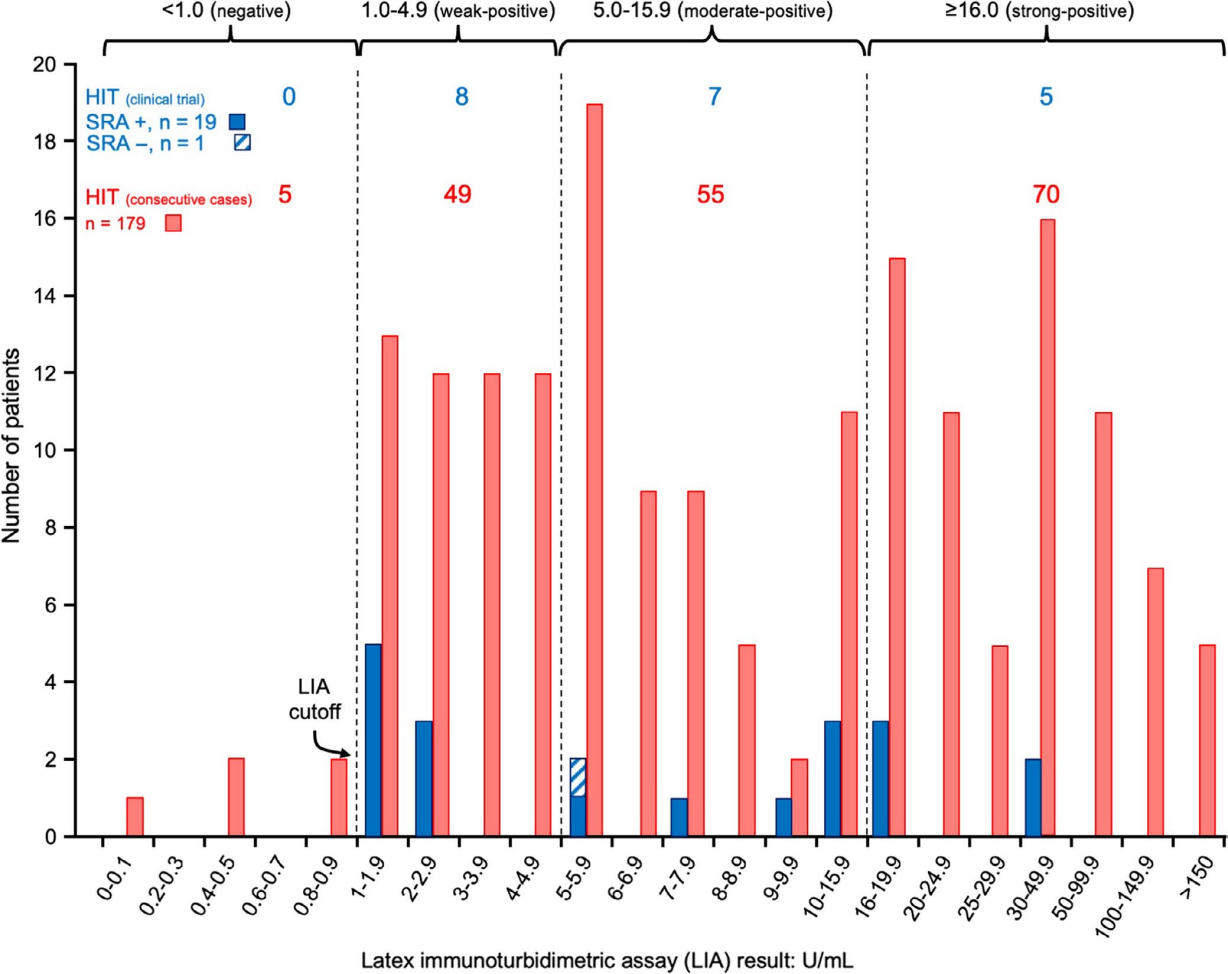
Comparison of LIA reactivities in 20 HIT patients in hip replacement surgery thromboprophylaxis clinical trial vs hospital‐wide population of 179 consecutive patients with HIT diagnosed at one institution (Hamilton General Hospital). The hatched symbol indicates the single trial patient recognized with SRA‐negative HIT. Overall, there is a similar distribution of LIA reactivities between the two patient populations shown. HIT, heparin‐induced thrombocytopenia; LIA, latex immunoturbidimetric assay; SRA, serotonin‐release assay; U, units

Figure 4 shows the distribution of the positive LIA results for the 20 patients in our trial who either developed SRA seroconversion with an associated abnormal platelet count profile (classic, proportional, blunted), along with the single patient identified with SRA‐negative HIT. These reactivities are compared with 179 patients with HIT from the Hamilton General Hospital who were investigated by LIA in our laboratory. The median LIA reactivity was similar between the two patient populations: 7.1 (median; range, 1.2‐35.5) for the 19 patients with SRA‐positive HIT in the clinical trial vs 7.8 (median; range, 1.0‐214.9) for the 179 consecutive patients with SRA‐positive HIT recognized in our institution.

## 4 DISCUSSION

We describe a study of LIA seroconversions in patients with serial plasma samples obtained in a historic clinical trial of post‐hip replacement heparin thromboprophylaxis.^26^ We found that the LIA tested positive in all 19 SRA‐positive patients identified in our thromboprophylaxis trial for which available plasma allowed for LIA testing. This high test sensitivity is consistent with our previous evaluation of the LIA, in which we reported a sensitivity of 97.4% (152/156; 95% CI, 93.6%‐99.3%),^20^ and which remained similarly high after we extended our evaluation to a greater number of consecutive SRA‐positive patients identified at our institution, as shown in Figure 4 (174/179, 97.2%; 95% CI, 93.6%‐98.8%). We also found that the timing of LIA seroconversion was similar to that of the polyspecific EIA‐GAM, another PF4‐dependent immunoassay with high sensitivity for HIT. Moreover, for all evaluable patients, the LIA was already positive at (or shortly before) the onset of the HIT‐related platelet count fall, a finding that mirrors what we have reported for the EIA^11^ and SRA.^12^

The sensitivity of the LIA for HIT might be similar to that for the SRA, given that some patients with HIT may test SRA‐negative, that is, so‐called “SRA‐negative HIT.” Indeed, we identified such a patient in our study (Figure 3). This patient, who had a high 4Ts score and who tested EIA‐positive, demonstrated LIA seroconversion. Interestingly, this patient's LIA reached its maximal value (5.5) on the same day the platelet count began to fall. This patient also showed platelet count recovery with continued heparin exposure, a phenomenon that we have reported in some patients with HIT in whom heparin is continued.^33^ Our observations add further support to the transience of HIT antibodies and the potential for platelet count recovery to occur in some patients with HIT even when heparin is continued. Our identification of one patient with SRA‐negative HIT in a clinical trial that identified approximately 20 patients with HIT (depending on the definition of thrombocytopenia used) is consistent with the long‐standing view of our laboratory that our SRA has a sensitivity of approximately 95%,^34^ that is, a false‐negative test result is obtained in approximately 1 in 20 patients who have HIT.

Our study also confirms an observation we made in our previous evaluation of the LIA,^20^ namely that patients with HIT often have weak‐positive test results by LIA. In our 19 SRA‐positive patients evaluated in the LIA, we found that 8 (42.1%) had results within the “weak” category (1.0‐4.9 U/mL), with all eight yielding results between 1.0 and 2.9 U/mL. This finding is in marked contrast to results in the EIA, in which very few SRA‐positive patients (<5%) yield weak‐positive EIA results, defined as values between 0.40 and 1.00 OD units.^35^ It is important for clinicians to be aware of this important difference between the LIA and EIAs; otherwise, there is a risk of a clinician discounting a “weak” LIA test result as being unlikely to be HIT, whereas we found that approximately one‐third of patients with confirmed HIT had “weak” LIA results (between 1.0 and 4.9 U/mL).

In conclusion, our study evaluating LIA seroconversion profiles using archived plasma samples from patients with HIT identified in a historic post‐hip replacement surgery trial has shown that positive LIA test results are present at the onset of HIT‐related platelet count fall. Further, our study identified a single patient who met criteria for SRA‐negative HIT; this patient also demonstrated LIA seroconversion, indicating that LIA testing may be useful for evaluating patients suspected of having SRA‐negative HIT.

## CONFLICT OF INTEREST

Dr Warkentin reports having received consulting fees from Aspen Global and Octapharma; research support and consulting fees from WL Gore and Instrumentation Laboratory; royalties from Informa (Taylor & Francis); and consulting fees related to medical‐legal testimony. The remaining authors declare no competing financial interests.

## AUTHOR CONTRIBUTION

Theodore Warkentin designed the research study, Jo‐Ann Sheppard performed the LIA testing and summarized the results, and both wrote the first draft of the manuscript. James Smith performed the PF4‐enhanced SRA. Ishac Nazy and Donald Arnold oversaw the laboratory investigations and edited the manuscript. All authors reviewed and approved the final version of the manuscript.
